# Fanconi anaemia as a human model of accelerated epigenetic and immune ageing

**DOI:** 10.1016/j.arr.2026.103038

**Published:** 2026-03

**Authors:** Eunike Velleuer, Carsten Carlberg

**Affiliations:** a Department for Cytopathology, Heinrich-Heine-University Düsseldorf, Düsseldorf D-40225, Germany; b Department for Pediatric Hemato-Oncology, Helios Children’s Hospital, Krefeld D-47805, Germany; c InLife Institute of Animal Reproduction and Food Research, Polish Academy of Sciences, Olsztyn PL-10-683, Poland; d School of Medicine, Institute of Biomedicine, University of Eastern Finland, Kuopio FI-70211, Finland

**Keywords:** Epigenetic regulation, DNA repair, Immune ageing, Fanconi anaemia, Nutrigenomics

## Abstract

Fanconi anaemia (FA) is a DNA-repair disorder that compresses multiple hallmarks of ageing into childhood and early adulthood. Persistent genomic instability in FA precipitates oxidative stress, inflammatory remodelling, and metabolic reprogramming, which together erode epigenetic integrity and immune competence. Here we provide evidence FA-specific DNA-repair failure is linked to mitochondrial metabolism, nutrient-sensing networks, and immune dysfunction. In this context, we discuss how these interactions accelerate epigenetic drift and cancer susceptibility. We propose FA as a human “time-lapse” model to separate the sequence and interdependence of selected ageing hallmarks, such as genome instability, epigenetic deregulation, stem cell exhaustion, and immunosenescence, which together contribute to a markedly increased risk of early cancer development. We further highlight nutrigenomic mechanisms, including vitamin D-dependent chromatin remodelling and redox-sensitive cofactors, that modulate epigenetic states and immune resilience. Framing FA within the broader framework of ageing biology suggests testable biomarkers and precision-prevention strategies aimed at stabilising the epigenome, delaying carcinogenesis, and prolonging healthspan.

## Highlights

• **Compression of ageing hallmarks** like genome instability, epigenetic drift and immunosenescence into early life.
• **Persistent DNA damage** couples to metabolic/mitochondrial stress to reshape the epigenome and immune function.
• **Nutrient-sensing and redox-dependent cofactors** (e.g., NAD⁺, α-ketoglutarate, vitamin D) modulate chromatin resilience.
• **Providing a “time-lapse” human model** to benchmark biological-age biomarkers and test preventive strategies.
• **Extending conceptual and translational implications** to therapy-induced ageing and population cancer risk.

## 1 Introduction

**Fig. 1:**
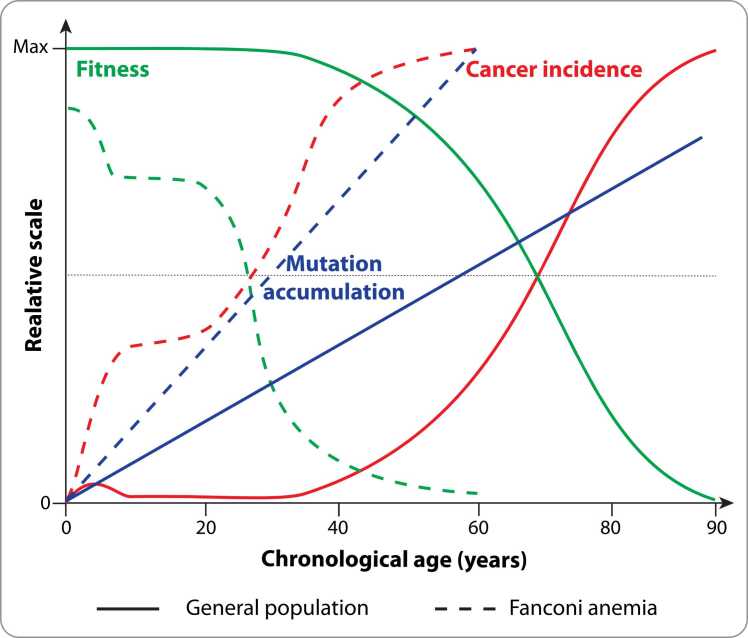
Compressed timeline of ageing and cancer onset in FA. Conceptual schematic comparison of mutation accumulation, cellular fitness, and cancer incidence trajectories between individuals with FA and the general population across chronological age. In FA, defective DNA repair leads to accelerated genomic instability and epigenetic deterioration, resulting in a rapid decline of cellular fitness and early cancer onset. This compression of molecular and ageing-associated processes into the first two decades of life exemplifies premature ageing in FA.

Ageing emerges from interconnected hallmarks like genome instability, epigenetic alterations, mitochondrial dysfunction, and immune decline that usually unfold over decades (Lopez-Otin et al., 2023b). Rare disorders that accelerate specific hallmarks provide leverage to resolve their sequence and interdependence. FA, a DNA-crosslink-repair defect, concentrates these processes into early life and therefore offers an informative human model of accelerated and premature manifestation of ageing hallmarks arising from a specific DNA interstrand crosslink repair defect (Velleuer and Carlberg, 2024). The syndrome exemplifies multiple hallmarks of ageing within the first two decades of life (Fig. 1).

**Fig. 2:**
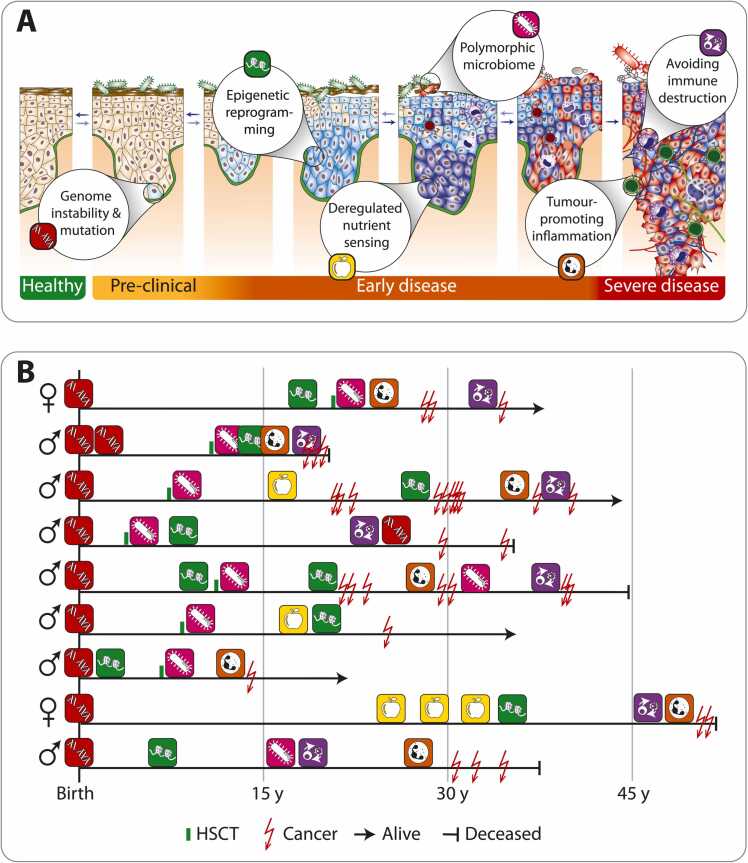
Sequential emergence of ageing hallmarks and cancer development in FA. Conceptual representation of the progressive biological transitions underlying tumourigenesis (A). Defective DNA repair induces genome instability, which triggers epigenetic reprogramming, metabolic dysregulation, and immune evasion. These interconnected processes drive chronic inflammation and ultimately cancer development, recapitulating the sequential emergence of ageing hallmarks, but occurring decades earlier in FA. Schematic representation of individual clinical trajectories in FA (B). The diagram illustrates qualitative timelines of disease progression in selected patients, including the timing of HSC transplantation, cancer occurrence, and survival outcomes. Symbols indicate key clinical events and transitions rather than quantitative or proportional time intervals. The figure is intended to visualize inter-individual variability and the compressed manifestation of selected ageing-associated hallmarks in FA, not to depict chronological ageing or population-based survival curves.

FA is caused by inherited defects in the FA/BRCA DNA repair pathway, resulting in hypersensitivity to DNA crosslinks, genomic instability, bone marrow failure, and an increased predisposition to cancer (Alter, 2017, Dufour, 2017, Brosh et al., 2017, Velleuer and Dietrich, 2014). This compressed manifestation of ageing hallmarks parallels recent efforts to identify biomarkers that quantitatively capture biological age beyond chronological time (Loughlin et al., 2025). The sequential emergence of these hallmarks mirrors the “somatic evolution” of cancer, where genomic instability alone is insufficient for malignancy, and a progressively deteriorating microenvironment that alters clonal selection becomes a critical driver (Fig. 2A).

FA patients display a constellation of features that parallel premature biological ageing, such as stem cell exhaustion, endocrine dysfunction, chronic inflammation, and early-onset cancer (Brosh et al., 2017). Persistent DNA damage and replication stress in FA trigger metabolic and immune remodelling that recapitulate core ageing pathways, including oxidative stress, and epigenetic drift. In this review, we synthesize current evidence linking genome instability, metabolic reprogramming, and immune dysfunction in FA, and discuss how these converging mechanisms illuminate core principles of ageing biology and the accelerated manifestation of ageing hallmarks, providing a framework for targeted nutritional and therapeutic interventions. Accordingly, FA should not be interpreted as a model of physiological ageing, but rather as a genetically defined disorder in which selected ageing hallmarks emerge prematurely as secondary consequences of defective DNA damage repair. This metabolic vulnerability provides a mechanistic entry point for nutrigenomic modulation, as dietary inputs can directly influence mitochondrial function, inflammatory tone, and epigenetic regulation in FA.

## 2 FA and the Hallmarks of Ageing

Ageing biology involves interconnected hallmarks, including genomic instability, telomere attrition, epigenetic drift, deregulated nutrient sensing, mitochondrial dysfunction, cellular senescence, stem cell exhaustion, and impaired intercellular communication (Lopez-Otin et al., 2023a). Although these features typically accumulate over decades, many emerge strikingly early in individuals with FA, effectively compressing the timeline of selected ageing hallmarks into childhood and adolescence (Fig. 1). The molecular hallmarks detected in FA align closely with recently established biomarkers of ageing in humans and non-human primates (Wu et al., 2025), reinforcing the view that FA constitutes an accelerated, genetically encoded manifestation of selected conserved ageing-associated trajectories.

Accumulating evidence indicates that genomic instability is the initiating hallmark of FA and orchestrates the emergence of subsequent ageing- and cancer-enabling traits. Longitudinal patient data (Fig. 2**B**) illustrate that, although the order of secondary hallmarks may vary, genomic instability consistently precedes tumourigenesis, offering a uniquely structured model of early cancer evolution seldom accessible in the general population. In FA, genetic and epigenetic alterations co-evolve under persistent microenvironmental stress, highlighting how chromosomal instability interacts with epigenetic plasticity to shape cancer susceptibility (Zhang et al., 2024).

Although chromosomal instability occurs systemically in FA, cancer susceptibility is not universal (Garcia-De-Teresa et al., 2020). Elevated risk is confined mainly to the bone marrow and to epithelial surfaces of the upper and lower aerodigestive tract. Cancers of the head and neck derived in FA individuals display a much higher rate of structural variants compared with those from the general population. Moreover, DNA aneuploidy was identified as the point of no return in the tumourigenesis of those epithelial cancers in FA (Velleuer et al., 2020). However, this tissue selectivity of tumourigenesis in FA emphasizes that genomic instability alone cannot drive malignancy. This distinction emphasizes that accelerated ageing and cancer are mechanistically linked but biologically separable processes. Rather, additional hallmarks like epigenetic dysregulation, metabolic stress, and immune erosion, act in concert to determine cancer susceptibility in FA.

Clinical and experimental studies show that tissue-specific vulnerability in FA reflects interactions among genomic instability, metabolic dysregulation, and immune erosion (Velleuer and Carlberg, 2024). These features parallel ageing-associated mechanisms in the general population and may explain why cancer risk escalates so early in selected tissues. Together, this triad of genome instability, metabolic dysregulation, and immune erosion exemplifies the convergent forces driving premature cancer development.

Mechanistically, bone marrow niche biology illustrates that haematopoietic stem cell (HSC) maintenance critically depends on a hypoxic, metabolically regulated microenvironment (Kemna et al., 2025). In this setting, quiescent long-term HSCs rely predominantly on anaerobic glycolysis while suppressing mitochondrial oxidative phosphorylation to minimize reactive oxygen species (ROS) generation. Disruption of this metabolic equilibrium by chronic DNA-damage stress, inflammation, or impaired autophagy compromises HSC fitness and accelerates exhaustion. In FA, persistent genotoxic stress and mitochondrial dysfunction likely precipitate a premature metabolic shift from glycolytic to oxidative states, thereby promoting stem cell attrition and early immunosenescence (Brosh et al., 2017).

FA and therapy-induced ageing share striking molecular parallels, including chronic DNA damage, mitochondrial dysfunction, and systemic inflammation (Demaria, 2025). In both contexts, accelerated biological ageing and erosion of tissue homeostasis are prominent. These analogies suggest that FA recapitulates the long-term adverse effects of cancer therapy on an intrinsic, genetically programmed timescale.

Together, these findings position FA as a unique clinical model for dissecting the interplay among genome maintenance, metabolism, and immune function that defines biological ageing.

## 3 Epigenetic programming in FA

**Fig. 3:**
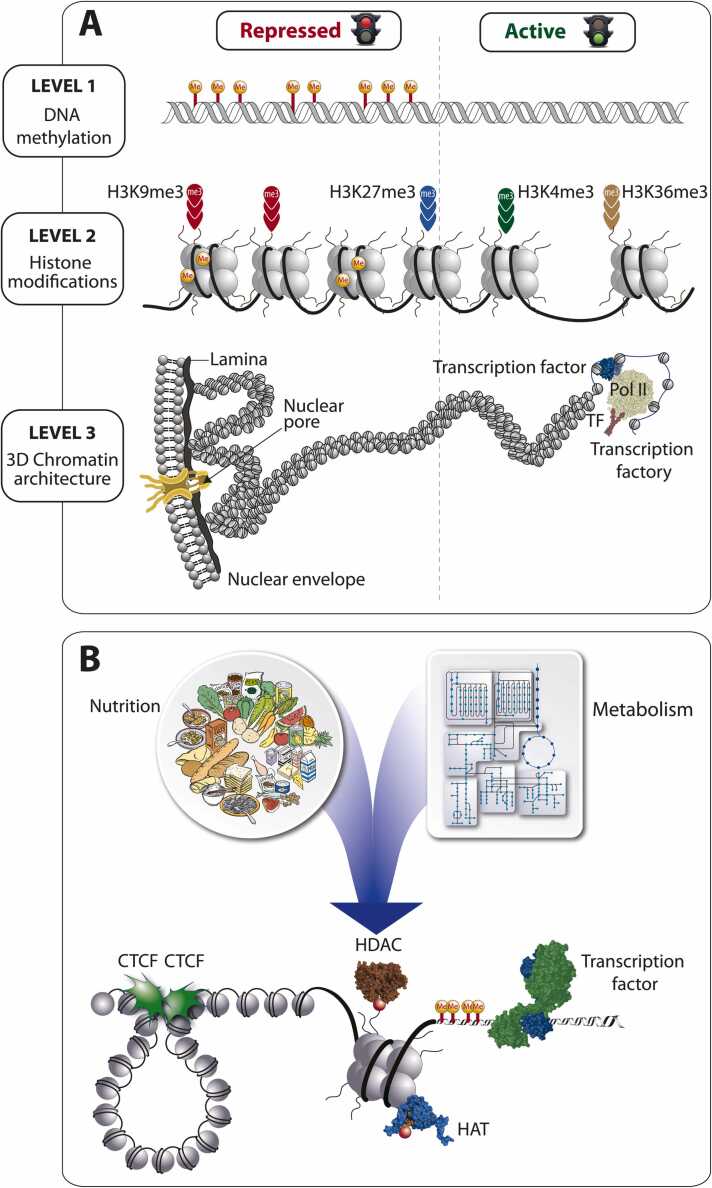
Epigenetic regulation as a multilayered interface between metabolism, chromatin architecture, and gene expression. The epigenome comprises three hierarchical layers of regulation: DNA methylation, histone modifications, and higher-order 3D chromatin organization (A). Nutrient- and metabolite-derived cofactors such as vitamin D, S-adenosylmethionine (SAM), NAD⁺, and acetyl-CoA dynamically modulate these layers through the regulation of transcription factors (e.g., VDR) and chromatin-modifying enzymes including histone acetyltransferases (HATs) and histone deacetylases (HDACs) (B). Together, these mechanisms link nutrition and metabolism to transcriptional regulation and epigenetic resilience.

The epigenome operates through three interconnected regulatory layers that orchestrate gene expression and cellular identity without altering the underlying DNA sequence (Carlberg, 2024) (Fig. 3**A**). DNA methylation stabilizes transcriptional repression, histone modifications fine-tune enhancer and promoter activity, and three-dimensional (3D) chromatin organization governs long-range regulatory interactions. Together, these layers constitute a molecular memory system that preserves developmental identity while remaining dynamically responsive to environmental and metabolic signals.

In FA, repeated recruitment of repair and chromatin-remodelling complexes at stalled forks can misallocate writers/erasers, converting transient DNA-damage responses into stable methylation and histone-mark changes that rewire gene regulation. This leads to aberrant epigenetic modifications that destabilize transcriptional networks, impair differentiation programs, and increase susceptibility to cancer development (Garcia-De-Teresa et al., 2020). Recent epigenomic analyses of FA models and patient-derived cells reveal aberrant methylation landscapes and disrupted chromatin topology that correlate with differentiation defects and malignancy risk (Pagliara et al., 2023). Non-mutational epigenetic reprogramming is therefore a central hub linking repair failure to inflammation, microbiome shifts, and metabolic stress (Zhu et al., 2021). Consequently, the FA epigenome represents an active interface translating DNA-damage signalling into altered cell fate and tumourigenic potential.

Epigenetic reprogramming can serve as a early and permissive driver of tumour initiation rather than a downstream consequence (Zhang et al., 2024). For example, hypermethylation of tumour-suppressor loci or disruption of chromatin topology, such as loss of insulator function *via* CTCF (CCCTC-binding factor) site methylation, can generate oncogenic enhancer–promoter interactions independent of mutation load (Carlberg and Velleuer, 2021). Comparable processes are likely operative in FA, where chronic DNA-damage signalling fosters epigenetically vulnerable chromatin states that facilitate clonal expansion and malignant evolution.

**Fig. 4:**
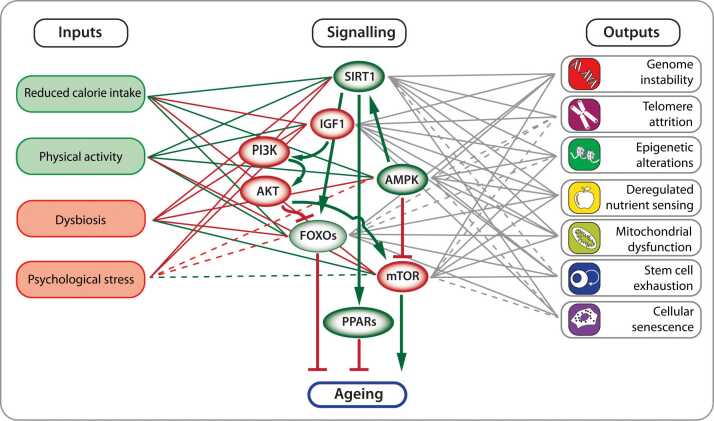
Hallmarks of ageing and modulatory pathways linking environmental inputs to cellular outcomes. Schematic overview illustrating how key environmental and lifestyle factors, including reduced calorie intake, physical activity, dysbiosis, and psychological stress, modulate major signalling pathways such as PI3K–AKT, IGF1 (insulin-like growth factor 1), mTOR, FOXOs, AMPK, SIRT1, and PPARs. These pathways integrate nutritional and metabolic signals to control cellular processes central to ageing, including genome instability, epigenetic alterations, telomere attrition, mitochondrial dysfunction, stem cell exhaustion, deregulated nutrient sensing, and cellular senescence. SIRT1-mediated deacetylation of FOXO transcription factors and its role in stress resistance and metabolic regulation are indicated.

From a nutrigenomic perspective, diet and metabolism directly regulate epigenetic networks (Klotz and Carlberg, 2023). Core nutrient-sensing pathways, including AMPK (AMP-activated protein kinase), SIRT1 (sirtuin 1), FOXO (forkhead box O transcription factor), and mTOR (mechanistic target of rapamycin), integrate cellular energy status with chromatin regulation (Wu et al., 2022) (Fig. 4). These circuits operate under strict redox control: sirtuin activity depends on NAD⁺ availability, while chromatin-modifying enzymes such as TETs (ten-eleven translocation dioxygenases) and lysine demethylases require α-ketoglutarate and ascorbate as cofactors (Klotz and Carlberg, 2023). Accordingly, nutritional interventions that modify the redox milieu, such as caloric restriction or polyphenol supplementation, can recalibrate epigenetic states. This redox–epigenetic coupling provides a mechanistic explanation for how nutrient status influences cellular ageing and carcinogenesis, suggesting that targeted dietary or pharmacological modulation may enhance epigenetic resilience in FA.

Within the bone marrow niche, metabolic pathways such as HIF1 (hypoxia-inducible factor 1)–driven glycolysis, PPAR (peroxisome proliferator-activated receptor)-mediated fatty-acid oxidation, and the PI3K (phosphoinositide 3-kinase)–AKT (protein kinase B)–mTOR axis form an integrated regulatory network that couples energy availability to chromatin remodelling and differentiation capacity (Kemna et al., 2025) (Fig. 4). Perturbations of these circuits through oxidative stress, lipid imbalance, or inflammatory cytokines can directly reshape the FA epigenome by altering metabolite pools that serve as cofactors for DNA and histone-modifying enzymes (Zhu et al., 2021). Incorporating metabolic regulation into FA models of epigenetic drift may therefore clarify how microenvironmental stress translates into heritable transcriptional reprogramming.

These findings emphasize the importance of considering epigenetic reprogramming as both a biomarker and a mechanistic driver of premature cancer development.

## 4 Nutritional and metabolic modulation of the epigenome

Among modifiable environmental influences shaping the epigenome, nutrition exerts particularly profound and multifaceted effects (Burton and Lillycrop, 2019). Dietary components not only supply substrates for chromatin modification but also deliver signalling molecules that activate nuclear receptors (Zhao et al., 2019) (Fig. 3**B**). Vitamin D provides a paradigmatic example: its active form, 1,25-dihydroxyvitamin D₃, binds the vitamin D receptor (VDR) to induce chromatin remodelling at hundreds of genomic loci (Carlberg et al., 2023). In myeloid cells, VDR activation controls gene networks governing differentiation, apoptosis, cytokine production, and antimicrobial defence. These processes are vital for maintaining epithelial and immune homeostasis. Epigenetic regulation of vitamin D target genes such as *CYP24A1* (cytochrome P450 family 24 subfamily A member 1) and *HSD11B2* (11β-hydroxysteroid dehydrogenase type 2) in the placenta links maternal nutrient status to offspring immune and metabolic programming (Basak et al., 2024, Mazur et al., 2022). These findings are consistent with the hypothesis that adequate vitamin D status could enhance epithelial and immune resilience in FA although interventional evidence remains limited (Velleuer and Carlberg, 2020). The overlap between vitamin D–responsive pathways and FA-related vulnerabilities raises the possibility that optimal vitamin D status could mitigate carcinogenic risk (Bilani et al., 2021).

Lifestyle factors, such as diet, pathogen exposure, and immune-modulating therapies, can shift the balance between epithelial health and dysplasia (Sun et al., 2024). Such insights point to the value of integrating nutrigenomic data streams into predictive models of FA cancer progression. Interestingly, inflammatory and metabolic signals constitute key “extrinsic driver events” that cooperate with intrinsic lesions (Zhang et al., 2024). Nutritional inputs, through their modulation of oxidative stress, cytokine signalling, and chromatin accessibility, may thus functionally resemble the environmental modulators of early tumourigenesis.

The gut microbiome serves as an important intermediary linking nutrition to epigenetic and immune homeostasis (Paul et al., 2015). Diets enriched in fibre, polyunsaturated fatty acids, and fermented foods favour *Faecalibacterium* and other taxa that produce antiinflammatory short-chain fatty acids and associate with reduced epigenetic age. Conversely, dysbiosis dominated by *Ruminococcus* species correlates with chronic inflammation and “inflammageing” (Hall et al., 2017). Recent studies in nutritional epigenomics provide mechanistic support that these diet–microbiome–epigenome interactions may be particularly relevant in FA, where persistent inflammation and defective autophagy amplify systemic stress (Rodriguez et al., 2021).

Additional nutritional modulators, including plant-derived polyphenols, dietary restriction, and pharmacological mimetics such as metformin, activate energy- and stress-sensing pathways that converge on the epigenome (Madeo et al., 2014). By rebalancing the interplay between senescence, differentiation, and transformation, these inputs provide mechanistic links between diet and cancer resilience in FA. Consistent with broader nutri-epigenomic models, dietary lipids (e.g., omega-3 fatty acids) and polyphenols influence histone acetylation and non-coding RNA expression at inflammatory loci (Del Saz-Lara et al., 2022). In the context of FA, such nutrigenomic pathways may be central to maintaining redox balance and chromatin stability. This illustrates how diet and metabolism shape epigenetic resilience (Yang et al., 2023), supporting diet-based strategies to mitigate epigenetic vulnerability in FA.

Recent multi-omics profiling of an exceptionally long-lived individual revealed that sustained consumption of fermented dairy products fostering *Bifidobacterium* growth coincided with a rejuvenated microbiome and a markedly younger epigenetic age despite advanced chronological age and telomere attrition (Santos-Pujol et al., 2025). Although derived from a single individual, these observations are consistent with population-based evidence linking microbiome composition, inflammation control, and epigenetic stability to extended healthspan. We emphasize that this study does not define general mechanisms of ageing, but rather illustrates how coordinated regulation of multiple ageing hallmarks may support exceptional healthspan. This demonstrates that diet-driven microbial ecology can modulate systemic inflammation, mitochondrial function, and DNA-methylation maintenance. These are the same mechanistic axes that are perturbed in FA.

## 5 Accelerated Immune Ageing in FA

Immune surveillance represents a primary defence against cancer development (Tufail et al., 2025). Its effectiveness depends on a balanced repertoire of naïve and effector T cells, cytotoxic natural killer cells, competent antigen-presenting cells, and the absence of chronic inflammation. In FA, early immune dysfunction occurs: bone marrow failure limits immune cell regeneration, while persistent DNA damage depletes haematopoietic progenitors and perturbs differentiation (Ceccaldi et al., 2012). Within the bone marrow niche, inflammatory cytokines such as IFNγ, TNF, and IL1β redirect HSC metabolism from glycolysis to mitochondrial respiration, increasing ROS production and accelerating exhaustion (Kemna et al., 2025). This inflammatory-metabolic coupling has been proposed as a mechanistic bridge linking chronic genotoxic stress to premature immune senescence in FA.

This inflammatory-metabolic coupling provides a plausible bridge from chronic genotoxic stress to premature immunosenescence. Disrupted epigenetic programming worsens immune defects, as haematopoietic differentiation depends on chromatin landscapes that integrate nutritional and hormonal signals (Buenrostro et al., 2018). Loss of epigenetic flexibility undermines immunocompetence, allowing emerging malignant clones to evade surveillance (Carlberg and Velleuer, 2024). The resulting early immune ageing mirrors the inflammageing typical of advanced age but manifests decades earlier in FA, leading to impaired immune surveillance and increased susceptibility to malignant transformation. In this context, cancer represents a downstream clinical consequence of accelerated ageing-associated dysfunction rather than an intrinsic component of the ageing process itself. Similar immune exhaustion is seen in cancer survivors, where therapy-induced DNA damage and cellular senescence perpetuate chronic inflammation (Demaria, 2025). In both scenarios, persistent genotoxic and metabolic stress progressively erode epigenetic resilience, establishing a mechanistic continuum between DNA-repair failure, immune dysfunction, and early tumourigenesis. These insights emphasize the potential of nutritional and lifestyle interventions to restore immune balance and delay cancer development (Zhu et al., 2021).

At the systemic level, immune decline in FA recapitulates conserved ageing signatures observed across primate immune systems, including chronic activation of pro-inflammatory cytokines and metabolic reprogramming toward oxidative states (Wu et al., 2025). These parallels reinforce the concept that FA compresses canonical immune ageing trajectories into an accelerated timescale.

Integrative multi-omics analyses of extreme longevity indicate that preserved immunocompetence depends less on cumulative mutation burden and more on maintaining low-grade inflammation, efficient lipid metabolism, and resilient epigenetic control of immune gene networks (Santos-Pujol et al., 2025). In FA, where chronic genotoxic stress drives inflammatory exhaustion, these findings point toward dietary and probiotic strategies aimed at restoring metabolic–immune coupling and mitigating premature immunosenescence.

Nutrigenomic evidence reinforces the notion that dietary and lifestyle signals not only shape the epigenetic programming of immune cells but can also enhance surveillance against early neoplastic transformation in FA. Computational models of FA tumourigenesis highlight the close interdependence between immune surveillance, epithelial barrier integrity, and microbiome composition (Velleuer et al., 2023). This systems-level perspective argues for integrating immune monitoring into epigenetic analyses, recognizing immune dysfunction as both a biomarker and a mechanistic driver of early malignant transitions.

Chronic inflammation and immune remodelling mark decisive inflection points in early tumour evolution (Zhang et al., 2024). Immune dysregulation, arising from ageing, DNA-repair deficiency, or environmental stress, acts as a selective force shaping the survival and expansion of transformed clones (Yamashita et al., 2020). From an ecological perspective, this selective pressure mirrors adaptive landscapes in which immunosenescence and stromal remodelling create permissive niches for mutant outgrowth (Tien et al., 2023). In FA, persistent DNA damage and inflammation likely accelerate this sequence, effectively reproducing the immune–stromal decline typical of late-life tissues. Accelerated immunosenescence in FA can thus be viewed as a microenvironmental driver that parallels the inflammation-driven clonal evolution observed in general tumourigenesis (Liu et al., 2025).

Collectively, these findings integrate FA within a broader framework of immune ageing, illustrating how chronic DNA-damage signalling, metabolic stress, and loss of epigenetic flexibility converge to erode immunocompetence

## 6 FA as a Time-Lapse Model of Accelerated Ageing Hallmarks

FA provides a natural time-lapse model for investigating how selected hallmarks of ageing evolve and interact within a compressed lifespan. Recent efforts to define multilevel biomarkers of ageing, spanning cellular, tissue, and organismal levels, provide a framework for quantifying biological age beyond chronological time (Wu et al., 2025). Within this framework, FA could serve as a natural “stress test” for such biomarkers, enabling the identification of early molecular inflection points that precede cancer onset.

**Fig. 5:**
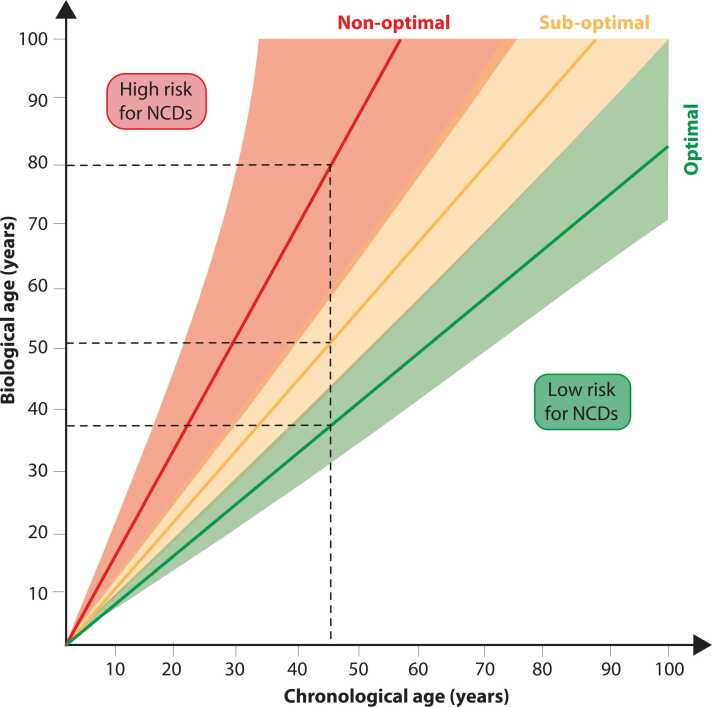
Divergence between chronological and biological age and its impact on disease risk. Schematic representation of the relationship between chronological age, biological age, and risk for non-communicable diseases (NCDs) like cancer. Individuals following optimal biological ageing trajectories maintain low NCD risk despite chronological ageing, whereas non-optimal or sub-optimal trajectories reflect accelerated biological ageing and elevated disease susceptibility. At a chronological age of 45 years (dashed line), an optimally ageing individual may exhibit a biological age of ∼38 years, a sub-optimal ager ∼51 years, and a non-optimal ager up to 80 years, illustrating how lifestyle and nutritional factors modulate biological ageing trajectories.

In the general population, genome instability and epigenetic drift gradually erode resilience after midlife, when cancer incidence rises (Lopez-Otin et al., 2023b). In FA, these processes are compressed into childhood and adolescence, with carcinomas like squamous cell carcinomas (SCCs) appearing in early adulthood (Alter et al., 2018). These clinical observations indicate that hallmark emergence is not strictly time-locked but reflects failure of genome repair and epigenetic maintenance, implying that biological ageing can, in principle, be modulated. A recent concept frames diet as a molecular regulator of ageing, acting through inflammation, the microbiome, and systemic resilience (Carlberg et al., 2025). Integrating this nutritional perspective with the FA model highlights how defective genome maintenance may interact with dietary and microbiome-derived signals that shape the trajectory of biological age (Fig. 5).

Extending these observations to FA supports the concept that accelerated epigenetic ageing is a unifying hallmark across genome instability syndromes (Kabacik et al., 2022). Conversely, exceptional longevity cases show that biological ageing can decelerate when metabolic and inflammatory homeostasis are preserved (Santos-Pujol et al., 2025). Multi-omics of a supercentenarian provides evidence for robust mitochondrial function, youthful methylome profiles, and antiinflammatory lipid metabolism illustrates that epigenetic and metabolic plasticity can buffer against genomic wear. This conceptually mirrors the inverse of FA biology.

The notion that genetic mutations alone are insufficient to explain early carcinogenesis aligns with the emerging “multi-driver” model of tumour initiation (Zhang et al., 2024). This highlights the integration of chromosomal instability, epigenetic reprogramming, and tissue context. In FA, the convergence of these forces occurs decades earlier, offering a compressed experimental system to study these transitions *in vivo*. This mirrors therapy-induced ageing in cancer survivors, where genotoxic stress drives similar molecular deterioration (Demaria, 2025, Guida et al., 2019). Thus, FA models therapy-induced ageing, illuminating shared molecular mechanisms such as DNA repair collapse, senescence, and mitochondrial decline.

Accordingly, FA offers a human model to test hypotheses about the temporal sequence of ageing hallmarks and to evaluate how environmental modulation may alter biological age trajectories.

## 7 Translational and computational outlook

Although FA is rare, the mechanisms it illustrates have broad relevance. In the general population, most cancer risk cannot be explained by inherited variants alone (Lappalainen et al., 2024). Much of the missing heritability likely resides in environmentally induced epigenetic modifications. Accordingly, early-life epigenetic imprints may disproportionately influence later cancer susceptibility.

Accelerated epigenetic clocks in FA highlight how biological ageing diverges from chronological age and provide a framework for developing biomarkers that reflect both the pace of ageing and responsiveness to interventions (Loughlin et al., 2025). Applying this biomarker concept to FA could identify which epigenetic and immune readouts best capture biological age acceleration. Integrating FA-derived multi-omic data into such benchmarking would validate FA as an extreme model for studying the causal architecture of biological ageing. The integration of FA-specific molecular data with standardized ageing biomarker panels (Wu et al., 2025) could help delineate which cellular and systemic parameters most accurately capture biological age acceleration. This harmonization would position FA within the broader landscape of translational biomarker research, bridging rare-disease biology and population-level ageing studies.

We suggest the following testable predictions:

• Metabolic–epigenetic coupling predicts that NAD⁺/redox interventions will measurably slow FA-specific methylation drift.
• Vitamin D_3_ supplementation with immunophenotyping will reduce FA inflammageing signatures.
• Microbiome modulation (fermented foods/fibre) will shift lipidomics and attenuate immune-age markers.

Contrasting FA with exceptional longevity reveals opposite poles of ageing-associated phenotypes: one marked by persistent genotoxic stress and inflammation, the other by metabolic efficiency, microbiome diversity, and a rejuvenated methylome (Santos-Pujol et al., 2025) (Fig. 5). Juxtaposing these extremes may uncover resilience biomarkers that predict the ability to maintain epigenetic stability under chronic stress. Similarly, comparisons with other progeroid disorders, such as Cockayne syndrome (CS), could help distinguish universal from disease-specific epigenetic ageing signatures, as CS methylomes overlap with normal ageing–associated CpGs (Crochemore et al., 2023).

Future progress will depend on longitudinal and integrative approaches combining multi-omics with functional assays tracking the epigenome, transcriptome, and immunocompetence of FA patients. This could identify early signatures of cancer development. Moreover, intervention studies with vitamin D_3_, dietary fibre or antioxidant-rich diets as well as physical activity may test whether lifestyle factors can delay cancer onset in FA individuals despite fixed DNA repair defects.

Framed within the evolutionary ecology of cancer, FA condenses the somatic evolution of malignancy into a compressed timescale, illustrating how genome instability and microenvironmental ageing jointly erode tissue integrity. Emerging computational modeling, including digital-twin and systems-simulation approaches, may help predict malignant transitions and identify early biomarkers of biological ageing in FA (Velleuer et al., 2023). FA cohorts, with well-defined molecular defects and predictable ageing trajectories, provide ideal datasets to calibrate such models against validated biomarkers of ageing. Combining longitudinal multi-omics with patient-specific simulations may ultimately allow early detection of malignant transitions and *in silico* testing of preventive strategies.

Mechanistically, these models span multiple scales, from differential equations describing signalling pathways to agent-based representations of tissue dynamics, and integrate single cell and spatial omics to preserve architectural context (Velleuer et al., 2023). Advances in generative transformer models further enable deep learning of temporal dependencies across large health datasets. Applied to rare diseases like FA, such approaches could model individual health trajectories as computational analogues of biological ageing, enabling virtual intervention testing and precision-prevention design.

## 8 Conclusions

Positioning FA within premature and accelerated ageing and nutrigenomics highlights that fixed DNA-repair defects interact with modifiable environmental inputs. This links rare-disease biology with population cancer risk and motivates precision-nutrition and systems-modelling approaches aimed at stabilising the epigenome, preserving immunocompetence, and extending healthspan.

Box 1 **Outstanding Questions.**

• How exactly does persistent DNA damage in FA rewire the epigenetic landscape: through stochastic drift, directed reprogramming, or selection of stress-resistant clones?
• To what extent are the immune and metabolic signatures of FA overlap with, or diverge from, those observed during physiological ageing?
• Can longitudinal multi-omics profiling of FA patients identify causal order among hallmarks like genome instability, epigenetic deregulation, and immunosenescence?
• Do nutritional or metabolic interventions (e.g., vitamin D, NAD⁺ precursors, or microbiome modulation) measurably attenuate epigenetic drift or inflammageing in FA?
• How can FA-derived models inform biomarker development for accelerated biological ageing in the general population?
• Could computational “digital twins” based on FA data predict individual ageing trajectories and therapeutic responses?

Box 2 **Limitations of the FA Model.**

While FA offers a powerful human model to study interconnected ageing processes, several limitations should be acknowledged:

• Restricted generalisability: FA represents an extreme and genetically homogeneous form of DNA-repair deficiency; its mechanisms may only partially overlap with those driving physiological ageing in the general population.
• Limited cohort size: The rarity of FA and clinical heterogeneity across genotypes constrain statistical power and make population-level extrapolation difficult.
• Confounding by treatment effects: Bone marrow transplantation, androgen therapy, and chronic inflammation can independently influence epigenetic and immune parameters, complicating causal inference.
• Scarcity of longitudinal and interventional data: Most available studies are cross-sectional, limiting insight into dynamic trajectories or the efficacy of potential preventive interventions (e.g., vitamin D, NAD⁺ boosters).
• Incomplete mechanistic resolution: Integrative multi-omics analyses linking DNA damage responses, chromatin modifications, and immune phenotypes are still lacking for FA.

These limitations highlight the need for longitudinal, multi-omics, and systems-modelling approaches that bridge rare disease biology with normal and therapy-induced ageing.

## CRediT authorship contribution statement

Conceptualization: CC. Writing – original draft: CC. Writing – review & editing: EV, CC. Visualization: EV, CC. Investigation (literature curation): EV. Funding acquisition: EV, CC.

## Funding

EV received funding from the Fanconi Cancer Foundation, the Deutsche Fanconi-Anämie-Hilfe e.V., 10.13039/501100001659Deutsche Forschungsgemeinschaft (DFG, project number 530200248) and the Elternverein Kinderkrebshilfe Krefeld e.V. CC is supported by the European Union’s Horizon Europe research and innovation program (grants numbers 952601 and 101119497) and the Polish National Science Centre (NCN, grant numbers 2023/49/B/NZ9/00402 and 2024/53/B/NZ2/00492).

## Declaration of Generative AI and AI-assisted technologies in the writing process

During the preparation of this work, the authors used ChatGPT (OpenAI) for language editing and readability improvements. After using this tool, the authors reviewed and edited the content as needed and take full responsibility for the final content.

## Declaration of Competing Interest

All authors declare no financial or non-financial competing interests.

## Data Availability

No new data were generated or analysed in this study.
